# Comparison of Self-Rated Health among Characteristic Groups of Vegetable Greenhouse Farmers Based on Exposure to Pesticide Residuals: A Latent Profile Analysis

**DOI:** 10.1155/2019/2518763

**Published:** 2019-04-04

**Authors:** Jiangping Li, Honghui Li, Shulan He, Min Xue, Danian Tian, Jian Zhou, Yongxin Xie, Huifang Yang

**Affiliations:** ^1^Department of Epidemiology and Health Statistics, School of Public Health and Management, Ningxia Medical University, Yinchuan 750004, China; ^2^Department of Occupational and Environmental Health, School of Public Health and Management, Ningxia Medical University, Yinchuan 750004, China; ^3^Department of Hygienic Chemistry, School of Public Health and Management, Ningxia Medical University, Yinchuan 750004, China

## Abstract

**Objective:**

The current study was aimed at using a latent profile analysis (LPA) model to classify greenhouse farmers into a potential cluster according to their exposure to pesticide residuals. Further, the association between self-rated health (SRH) and the cluster exposed to pesticide residual was explored.

**Methods:**

Four hundred sixty-four farmers from vegetable greenhouses were selected, their SRH information was gathered through questionnaires from the “Self-Rated Health Measurement Scale (SRHMS)” Version 1.0, and the corresponding pesticide residuals were detected in a laboratory. The linear mixed regression model was employed for association assessment.

**Results:**

Two latent clusters were extracted as samples, and the results showed that a high amount of pesticide residual accounted for poor physical health, but did not show statistical significance. In addition, an inverse significant association was observed between psychosocial symptoms and negative emotion and pesticide residual level. Furthermore, a diversity of significant relationship was observed in social health and its corresponding dimensions with latent cluster.

**Conclusions:**

LPA offers a holistic and parsimonious method to identify high-risk health clusters of greenhouse workers in various health aspects and allows for a personality-targeted intervention by a local health department.

## 1. Introduction

Agrochemicals are a homogeneity risk associated with various diseases resulting from occupational exposure [1–3]. The data from the Food and Agriculture Organization of the United Nations indicated that the volume of pesticide consumed severely increased from 2000 to 2016 in developing countries [4]. In addition, from 2008 to 2012, approximately 62% of pesticides were applied in the agricultural sector [5]. A close relationship was stated between agrochemical exposure and cardiovascular diseases [6], respiratory diseases [7–9], sleep disorder [10], Parkinson's disease [11], cancer, and tumor [12–14]. Unlike physical examination results, self-rated health (SRH) is generally considered as a valuable resource to assess the health condition on subjective information widely used in public health research and practices [15, 16]. In addition, SRH was recommended for population health monitoring by WHO [17] and is considered suitable for monitoring the health of greenhouse vegetable farmers in China. According to a group of special agricultural practitioners, the excessive exposure level of greenhouse farmers to pesticides could lead to degradation of their health, due to nonseasonal and hermetic running working conditions. Their health condition is of great concern because they play an important role in ensuring the daily supply of fresh vegetables to urban residents; therefore, specific risk factors must be evaluated and precision intervention could be applied for individuals.

Until recently, numerous studies have confirmed that the health condition of greenhouse farmers could be attributed to the single factor of occupational pesticide exposure by adjusting confounding variables by using the generalized linear approach. To reflect the association, most of these methods use a variable-centered approach, which does not consider individuals. Indeed, the variable-centered approach assumes individuals within a group as homogeneous, and the results are estimated based on the averaged indexes [18]. Latent profile analysis (LPA; or latent class analysis, LCA) is a specific case of a person-centred approach [19] that is used to identify individual profiles of participants according to the relationship pattern of the measured continuous variables. LPA is also a powerful technique that gleans insights into “hidden” characteristics to create profiles to provide better intervention for different exposure level of individuals.

The occupational exposure to pesticides affecting health is divided into synthetic and variant levels according to the condition of greenhouse farmers and their tendencies of pesticide usage. To the best of our knowledge, the synthetic effects of pesticide exposure to the health of individuals are not yet known in northwest China even through this knowledge could be important in a priori prevention of diseases and characteristic management of health. The present study was based in Ningxia, China, and was aimed at exploring the association between pesticides exposure/residual and self-rated health (SRH) used in a person-centered approach. The contributions of this paper to relevant literature are twofold: ensuring the number of latent clusters based on a farmer's personal features and identifying the association of latent clusters with SRH, and especially with corresponding health dimension.

## 2. Materials and Methods

### 2.1. Study Sample

Participants included a portion sample obtained from the* Pesticides exposure and cardiovascular disease survey in greenhouse vegetables farmers in Ningxia*, which is mainly supported by the Natural Science Foundation of China (NSFC) and was conducted in four greenhouse vegetable planting villages (Liangtian, Yinhe, Wudu, and Maosheng) from April to May in 2015, 2016, and 2017. The sample sites were located in Yinchuan City, Western China, which neighbors the Tengger desert, the fourth largest desert in China. In the investigation year for each village, one nonrepetitive (if one team was selected in this survey year, then this team was not considered in the sampling framework in the next survey year) resident team (primary resident unit in the village) was selected using a random sampling method. Then, the greenhouse farmers who met the inclusion criteria were invited to participate in the investigation.

The inclusion criteria for the participants were as follows. (1) The villager must be an adult of >18 years, who has resided at his/her current address for at least five years; (2) the villager was engaged in vegetable planting in greenhouses for more than one year; (3) the vegetable samples were allowed to be collected from their sheds; and (4) the farmers ate the vegetables grown in their greenhouses. Finally, 1368 villagers were obtained in the original design and 464 participants were satisfied condition of current study.

The study design and protocol were approved by the Medical Ethics Committee of Ningxia Medical University (No. 2014-090), and verbal consent was obtained from participants prior to the investigation.

### 2.2. Pesticides Residual Measurement

Villagers who participated in the survey and agreed to obtain vegetables from their sheds were considered for exposure to pesticide residuals. A quantity of 1.5 kg vegetables was collected using the plum blossom sampling method, which is a common sampling method in vegetable sample selection. Samples were separately packed into sealed bags, refrigerated at 4°C, and shipped to a laboratory to detect pesticide residual within 24 h. The vegetable samples were collected and tested according to the National and Agricultural Standards of China: GB/T14552-2003 [20] and NY/T 761-2008 [21], respectively.

#### 2.2.1. Pesticides Residual Detection in Vegetable Samples

Two categories were detected (cartbamate and organophosphorus pesticides) and ten types of pesticide residuals were monitored in our study: methomyl, propoxur, isoprocarb, fenobucarb, ethoprophos, disulfoton, parathion-methyl, fenitrothion, malathion, and chlorpyrifos.

#### 2.2.2. Sample Preparation

*(1) Sample Processing*. For preparing vegetable samples, each vegetable was cut into pieces, chopped in a juicer, and finally placed in a beaker for the next process.

*(2) Sample Extraction*. Samples of 20 g were weighed accurately, and 40 mL acetonitrile was added to homogenize the samples for 1 min by using a high-speed homogenizer. The filtrate was collected after the mixture was filtered and then transferred to a separating funnel. Sodium chloride was added to saturation and then allowed to stand at room temperature for stratification after violently shaking the funnel.

*(3) Sample Evaporation*. The organic-phase solution (upper layer) was accurately drawn into a round-bottom flask and then placed into a rotary to evaporate the organic-phase solution unit it was almost completely evaporated (water bath temperature ≤ 62°C).

(*4) Sample Purification*. A volume of 1:1 mixture of acetone and dichloromethane was used to configure the eluent. The activated CARB/NH_2_ column was rinsed with 5 mL of eluent and the flask was washed three times with 1 mL of the eluent; thus, the eluent was inhaled into the solid-phase extraction (SPE) column. After the liquid surface reached the surface of the SPE column, 12 mL eluent was added and 15 mL of the extract was collected. Washing liquid. Concentrated near dry. Finally, a constant volume of* n*-hexane was used for gas chromatography-mass spectrometry analysis.

### 2.3. Detection Conditions

The conditions of chromatography were as follows: helium was used as the carrier, with a column flow of 1.5 mL/min and injection volume of 1 *μ*L at the injection mode. In addition, splitless sampling was used and the shunt and spacer purge valves were opened after 1.5 min. The inlet temperature was 250°C and was initially programmed at 60°C, after holding for 2 min, at a speed of 10°C/min to 180°C, and finally at a speed of 3°C/min to 270°C.

The conditions of mass spectrometry were as follows. For selected ion monitoring, this study used the electron impact ionization method with an ionizing energy of 70 eV, ion source temperature of 230°C, transmission line temperature of 280°C, and a solution delay time of 12 min. Furthermore, the selected ions in each group were detected in the order of their outpeaks and time intervals.

The primary instrument model used in the experiment and its manufacturer are listed in Table S1, and the reagents and specifications used in the experiment are listed in Table S2.

### 2.4. SRH Measurement

SRH was measured using the questionnaire acquired from the* Self-Rated Health Measurement Scale (SRHMS) Version 1.0* developed by Xu and colleagues [15, 22–24]; the corresponding high reliability and validity were verified with respect to Chinese population, according to which the scale was a good alternative for reflecting the health status. SRHMS was developed considering the social, historical, and cultural factors. The questionnaire comprised 48 items divided into 9 dimensions and 3 subscales. Raw options ranging from 0 to 10 were marked on the horizontal axis and for participants assessed their scores on the line. B1, B2, and B3 were used to describe the physical symptoms and organic functions, daily physical activities, and physical mobility, respectively; M1, M2, and M3 were used to represent psychosocial symptoms and negative emotions, positive emotions, and cognitive functions, respectively; S1, S2, and S3 were used to depict role activity and social adaptability, social resources and social contacts, and social support, respectively. Three subscales, BZT (sum of B1 to B3), MZT (sum of M1 to M3), and SZT (sum of S1 to S3), represent the physical health, mental health, and social health, respectively. The total score of SRHMS, called SCZT, is summarized by these three subscales. Ten items needed to be converted owing to their inverse scores in the questionnaire (items 4, 5, 7, and 24-30). Further, items 18, 34, 46, and 48 were ignored in the calculation process of SCZT. In addition, the higher scores mean better health. The details of each dimension and subscale allocation plan have been represented in previous literature [15] and Table S3. The raw score was recalculated using the following formula in the final analysis strategy owing to different number of items in each dimension and for the intuitive contrast in our study.

(1) Transformed score=Actual  raw  score−lowest  possible  raw  scorePossible  raw  score  range×100

### 2.5. Covariates

Covariates were selected in our study according to the prior knowledge that was used in a lot of public health literatures [25]. Sociodemographic characteristics were proved to influence the SRH in Chinese [15, 26]. The following covariates were considered: number of family members, gender, ethnicity, age, education level, marital status, and income group. Behavior- and lifestyle-related variables, such as smoking status, drinking habit, physical exercise, self-rated salt consumption level, and breakfast-eating frequency, were also included. The presence of diseases in the survey period was measured by the question, “Do you have chronic diseases that were diagnosed by a medical doctor/health professional?”; this was answered simply as* Yes* or* No*.

### 2.6. Statistical Analysis

We decided to study the pesticide-residual profiles/clusters in the vegetable samples because local residents had high frequency of eating vegetable from their greenhouses; this was set as a direct predicator to reflect farmers' exposure pathway. The following 10 important indicators of pesticide residual were separately detected for LPA: methomyl, propoxur, isoprocarb, fenobucarb (first four pesticides are carbamates), ethoprophos, disulfoton, parathion-methyl, fenitrothion, malathion, and chlorpyrifos (all of last six are organophosphorus pesticides). The LPA was performed using Mplus version 7.4 (Linda Muthén & Bengt Muthén). LPA model is a specific form of LCA, in which the variance of the continuous index is decomposed into the intra- and intercategory variances according to the following equation:

(2) $$\begin{array}{c} \sigma_{i}^{2}=\overset{K}{\underset{k=1}{\sum }}P\left(\;c_{i}=k\;\right){\left(\;\mu_{ik}-\mu_{i}\;\right)}^{2}+\overset{K}{\underset{k=1}{\sum }}P\left(\;c_{i}=k\;\right)\sigma_{ik}^{2} \end{array}$$

where *μ*_*ik*_ and *σ*_*ik*_^2^ denote the mean and variance of index* i *in profile* k*, respectively, and *P*(*c*_*i*_ = *k*) is the latent profile probability. By assuming independence and homogeneity, the LPA model was transformed into the following expression [27, 28]:

(3) $$\begin{array}{c} f\left(\;y_{i}\;\right)=\overset{K}{\underset{k=1}{\sum }}P\left(\;c_{i}=k\;\right)f\left(\;y_{i}\;|\;c_{i}=k\;\right) \end{array}$$

where *y*_i_ is an object's score on a set of observed variables. Based on Bayes' theorem, the posterior probability was used to assign the observed cases to the* k* class as follows:

(4) $$\begin{array}{c} P\left(\;c_{i}=k\;|\;y_{i}\;\right)=\frac{P\left(c_{i}=k\right)f\left(y_{i}\;|\;c_{i}=k\right)}{f\left(y_{i}\right)} \end{array}$$

The best model was determined according to the procedure delineated by Nylund [29] and other previous studies [18, 28]. The following goodness-of-fit indicators were used to assess the best model: Bayesian information criterion (BIC), Akaike information criterion (AIC), sample-size adjusted BIC (a-BIC), and entropy. Among these indicators, smaller values of BIC, AIC, and aBIC indicate a better model, with the entropy ranging from 0 to 1, whereas higher values indicate better classification accuracy. A previous study confirmed that the model with an entropy ≥0.8 is adequate [18]. Moreover, according a simulation study [29] the use of BIC was reliable for confirming the latent class number for continues variables.

After controlling for covariates, a linear mixed regression model was used to explore the association between a pesticide-residual profile and SRH already assessed through the SRHMS questionnaire, including different dimensions, by using the STATA version 15.0 software (STATA Corporation, College Station, TX, USA). In the linear mixed regression model, the related expression is calculated as

(5) $$\begin{array}{c} y=X\beta +Zu+\varepsilon \end{array}$$

where *y* represents the *n* × 1 vector of responses, **X** denotes the *n* × *p* fixed-effect design matrix, *β* represents the fixed effects, **Z** is the *n* × *p* random-effect design matrix, *u* represents random effects, and *ε* represents the *n* × 1 vector of error [30]. Frequency and percentage were used to report categorical variables and mean ± standard deviation (SD) of the continuous variables. The *t*-test and *χ*^2^-test were conducted to estimate the differences between the covariates of profiles.

Seven models were employed to test the association between a pesticide-residual profile and SRH. Model 0 was an empty model, with the pesticide-residual profile as an independent variable. Model 1 was adjusted by variables of gender, ethnicity, age, education, marital status, and number of family members based on Model 0. Model 2 was created by adding an income group in Model 0 and Model 3 was created by adjusting variables of smoking status, drinking habit, physical excise frequency, breakfast-eating frequency, and salt in Model 0. Model 4 was created by adding the disease status in Model 0 and Model 5 was created by adjusting the year-of-survey. Finally, in Model 6, all potential confounders were considered; *P* < 0.05 was considered statistically significant. Subsequently, a stratified analysis was performed within the exposure time groups, and the full model was employed.

## 3. Results

### 3.1. Cluster of the Residual Pesticide

**Figure 1:**
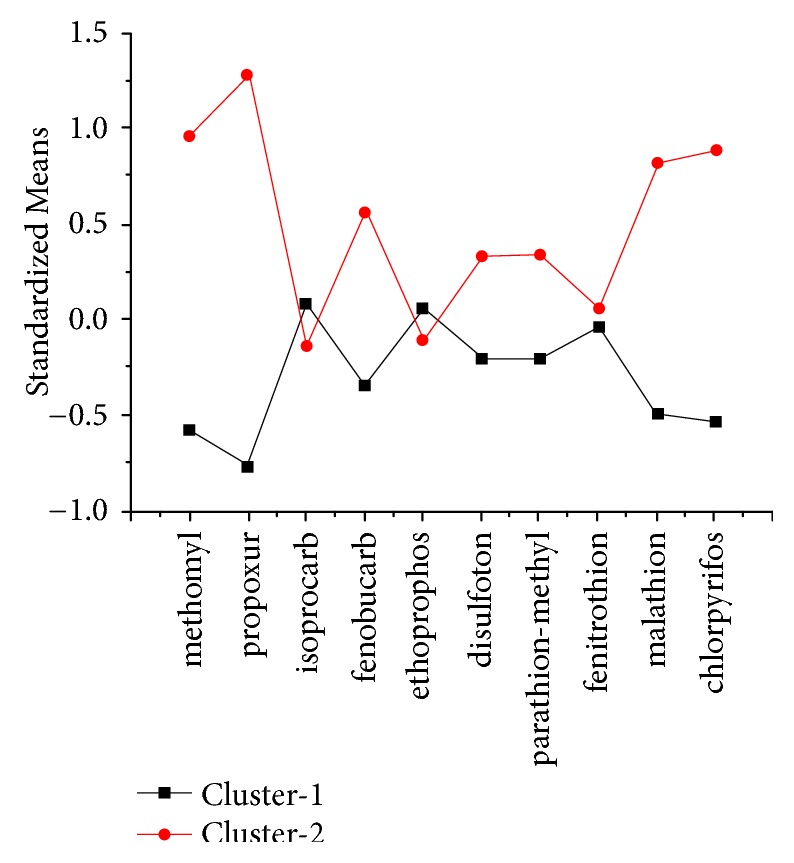
Cluster-specific probabilities of vegetable pesticide residual for the two-cluster model (n=464, farmers from vegetable greenhouse in Yinchuan, China).

In the current study, we considered cluster Models 1–4.  Table 1 presents the goodness-of-fit measure indexes. Cluster Model 3 appears to be more suitable than Models 1 and 2 because of high entropy and relatively low values of LL, BIC, aBIC, and AIC, while cluster 4 had lower entropy than the other three models. Theoretically, we should select three latent clusters; however, according to the category membership described in Table S4, the three latent clusters contain a smaller number of case-groups (only five participants were categorized into cluster 2). For illustration of which cluster model should be selected, Figures 1 and S1 show plots of cluster-specific probabilities, and as shown, the latent cluster cannot be easily distinguished by the curves of the three clusters (Figure S1). Next, we selected two clusters as the latent cluster number for vegetable samples. According to Figure 1, cluster 1 has a lower standardized mean of 10 pesticides; thus, cluster 1 is defined as a lower residual group; cluster 2 showed a higher standardized mean and was renamed as the higher residual group. Numerals 1 and 2 were used to represent clusters 1 and 2 in the analysis procedure. Finally, the two latent clusters memberships based on vegetable pesticide residuals were identified as comprising 290 (62.50%) and 174 (37.50%) farmers.

To verify the correction classification of two clusters, the rates of pesticide residues beyond national standard [31] between cluster 1 and cluster 2 were compared. The results showed that exceeding standard rate was higher in cluster 2 than cluster 1 for all kinds of pesticides except fenitrothion that exceeding standard rate in cluster 1 was 1.38% compared to zero in cluster 2. All of the above information showed that the definition for two clusters was appropriate.

### 3.2. Covariates Description and Difference between Clusters

In the present study, 462 farmers (99.57%) reported that they consumed vegetables derived from their greenhouse on a daily basis; two participants reported irregular consumption. Except the variables of gender, number of family members, education level, and breakfast-eating frequency, all of the remaining covariates showed statistically significant difference between the two latent groups (lower residual vs. higher residual,* P *< 0.05). The lower-residual cluster showed farmers with older age than the higher-residual cluster. Farmers of Hui ethnicity showed lower proportion in the lower residual cluster. In addition, the lower-residual cluster comprised higher proportion of married participants and low-income farmers. The detailed information is provided in Table 2.

### 3.3. SRH Description and Difference Test between the Clusters

Table S5 shows the raw and transferred scores in SRH along with different dimensions. The univariate analysis results showed that the psychosocial symptoms, negative and positive emotions, cognitive function, social resource, social contact, social support, and social health significantly differed between the clusters. Except psychosocial symptoms and negative emotions, the remaining five dimensions had a higher score in the higher-residual cluster, as detailed in Table 3.

### 3.4. Multiple Analysis for SRH

Although the multiple mixed linear regression results showed no significant association between clusters and total score of SRHMS in all the models, several dimension and subscale scores showed a close relationship with the cluster.

The association between vegetable-based pesticide-residual clusters and SRH is shown in Table 4. Some models of the results did not show robustness for all models. This means that the covariates would confound the relationship between clusters and SRH and would conclude the final results and discussion based on the full model. Unadjusted and adjusted models showed the association of psychosocial symptoms and negative emotions with residual clusters; the score of the psychosocial symptoms and negative emotions decreased 12.04 in the higher residual cluster compared to the lower residual cluster after controlling for all potential confounding factors. The subscale of mental health showed a significantly lower transfer score of 4.37 units in the higher-residual cluster compared to the lower-residual cluster. The average of role activity and social adaptability was lesser (3.69 unit) in higher latent cluster compared to that in the lower group. Further, social resource and social contact, social support, and social health showed a positive coefficient from lower to higher residual groups, that is, 5.77, 6.30, and 2.84, respectively. The residual cluster showed a stronger association with psychosocial symptom, negative emotion, social support, social resource, and social contact than with other factors. The effect size (coefficient) in the final model is bigger than those in other models in most dimensions.

### 3.5. Stratified Analysis

The stratified analysis was used to determine if the association differed for different exposure times. The results (data shown in Table S6) implied that there was no significant association for the physical health and pesticide residual level in exposure time stratification. The psychosocial symptoms of all stratified patients were negatively correlated with negative emotions and the residual group, and they were consistent with the results of all samples. The total scores of SRHMS did not show a significant association with the residual cluster for different exposure time level. This result is also in line with all sample results. For the dimensions of mental health, social resource and social contact, social support, and social health, there was no significant association in exposure time less than 200 days; however, the role activity and social adaptability showed a negative association in this stratum. There was no significant relationship between social resources, social contact, social support, social health, and residual clustering within 200 ~ 299 days; however, the scores of high pesticide residue group were significantly lower than those of the control group in terms of mental health, role activity, and social adaptability. There was a significant positive correlation between social resources and social contact, social support, social health, and residual clustering in stratification with an exposure time greater than or equal to 300 days. However, role activities had no significant relationship with social adaptability and mental health.

Overall, by the duration of pesticide exposure, the social health dimension and its subdimensions are closely associated. For the mental health dimension, significant associations were observed in the middle exposure time stratum. The different stratification results may be due to the small sample size effect. From Table S6, the width confidence interval appears to support the above hypothesis.

## 4. Discussion

This is the first study to use the proposed method to report on the field of occupational hazard exposure in greenhouse farmers to the best of our knowledge. We demonstrated individual pesticides typologies using LPA among greenhouse farmers and its association with self-rated health. And results showed negative relationship with “psychosocial symptom and negative emotion,” mental health, and “role activity and social adaptability,” and positive association with “social resource and social contact,” “social support,” and “physical health.”

Thus, in comparison to a physical examination result, SRH has been verified as a valuable predictor for reflecting health condition of individuals. Vegetable pesticide residuals were selected to assess the residual level in greenhouse farmers, most of whom (99.67%) daily consumed vegetables grown in their greenhouses; these depicted the routine exposure of farmers under specific pesticide circumstances and reflected the level of pesticide exposure directly in body, which is valuable information. Although several epidemiological studies have supported proof of association between occupational pesticide exposure and different single adverse health outcomes verified in a laboratory or clinically, they rarely assessed the association between an individual's pesticide-residual characteristic grouping and synthetic health condition. After extracting two latent profiles based on vegetable pesticide residual, greenhouse farmers were classified into two independent groups (lower- and higher-residual groups), and 174 (37.5%) individuals were categorized into the higher-residual latent profile. The study results did not show significant association of the synthetic effect of the overall detected pesticide residual with a population depicting lower SRH score; this is inconsistent with the results of clinical evidence [32]; the potential reason for this is that, among the middle age famers in our sample, there might exist the “health farmer” effect. Moreover, regarding the various subscales of health condition, the results showed that physical and mental health are negatively related to clusters but positively associated with social health. Further, the residual profile was not significantly associated with physical health; this could be due to the initial use of pesticides in the 2000s, the sale path was controlled and standardized by the Chinese government, and the farmers transitioned from the previous crude exposure to pesticides under abused conditions to long-term low dose exposure to them, and thus the cumulative body response might be delayed.

Lower scores were detected for psychosocial symptoms and negative emotions in the high pesticide-residual profile, indicating that a higher amount of pesticide residual would negatively impact psychosocial behavior. These results are in line with those from a previous study [33], which stated that high exposure level to pesticides would increase the incidence of depression, with respect to the samples obtained from the American Agricultural Health Study. Another study, through a cohort research design, explored the negative relationship between long-term exposure to herbicides and mental health in Canadian farmers [34]. This could have been because pesticide exposure interrupted the biochemical indices that regulate neuronal activity; these indices are mostly used as restraint control elements. Some other studies showed that enzyme activities (cholinesterase) in a pesticide-exposed group were statistically lower than in the control group [35, 36]. A potential explanation was the exposure associated with sleep problems including shortening of sleep duration and sleep apnea [37]. Sleep disorders in turn could potentially affect mental health.

Our research showed interesting results, with low scores for role activity, social adaptability in the high pesticide-residual profile but high scores for social resource and social contact, social support, and social health for the same residual profile when considering the final multivariable-controlled mixed regression model. Social health directly reflects the level of social capital [38]. A probable cause was that lower social capital might increase the chance of staying at a greenhouse or performing high intensity of physical work that supports more opportunities to consume greenhouse vegetable, because the lower social capital indicated that farmers had lower social interaction that would have more time staying in the greenhouses. The farmers' decision of planting larger areas of a greenhouse was plausible, and they need more social resource and social contact to help them sell their product; these demands could facilitate farmers in communicating and consolidating associations with villagers and peddlers. These behaviors could gradually accelerate the capacity of social support and social contact within farmers. In addition, vegetable trading compresses farmers' time for performing corresponding activities in communities; this explains the negative association between role activity and social adaptability with the residual profiles. In summary, pesticide residual associated with social health revealed complex characteristics, and the underlying mechanism needs to be further studied experimentally and through surveys to explore it. Stratified analysis results showed that different exposure time levels might be a predictor to influence self-rated health; however, a small sample size and wide confidence interval weakened the association, which requires larger samples and longitudinal data.

In the present study, all likely pesticide residuals were covered through the population-based retrospective survey in four villages of Yinchuan City, China, from 2015 to 2017. Unlike other classification methods, LPA is based on data features and showed categorization of each farmer objectively. LPA is termed as a probability method that has a potential advantage over distance cluster methods (such as* k*-means and hierarchical clustering) with lower misclassifications [28, 39]. Second, LPA has the ability to assess the impact of pesticide residuals in an integrated pattern and not in isolation [40]. Another strength of LPA was that the models allow adjusting of the covariates, keeping most of the information intact, allowing for the quantification of the uncertainty of class membership, and helping in the assessment of the goodness-of-fit [40]. Our results classified farmers into two independent latent clusters based on the presence of pesticide residual in occupational population; this could be a valuable method in the screening of high pesticide residual with respect to lower mental health score, the influence of which on social health showed diversity.

There are a few potential limitations of this research. First, the cross-sectional study design limits the causal inference between pesticide residual and health condition. Second, although most participants were reported to eat vegetables from their greenhouse on a daily basis, the study is biased in using the factor of vegetable pesticide residual to estimate residual level in the body. In addition, the data were obtained under the primary design that was focused on the relationship between prevalence of cardiovascular and pesticide behavior. Third, the study only focused on detecting two frequently used types of pesticides (cartbamate and organophosphorus); other exposure pathways also need to be assessed. This might considerably contribute toward assessing accumulative pesticide exposure. Further studies should gather blood samples to detect all likely exposure sources and conduct precise classifications and causal inferences based on a longitudinal study design.

## 5. Conclusions

Despite many limitations, this study provides a valuable method to determine synergistic effects of multiple pesticide-exposure sources in the occupational population. In conclusion, our findings cannot suggest the total SRH measured via SRHMS with respect to high pesticide residual. Regarding mental health, the results consistently showed low scores with respect to high residual profile; however, a diverse relationship was observed between social health and its dimensions. This personal-centered approach offers a holistic and parsimonious method for identifying the high risk in health clusters for greenhouse workers under various health aspects and offers a personality-targeted intervention by the local health department.

## Figures and Tables

**Table 1:** Goodness of fit measures of four different cluster models for vegetable pesticide residual.

Model	LL	BIC	aBIC	AIC	Entropy
1-Cluster	-6587.87	13280.54	13217.06	13197.74	1.000
2-Cluster	-5136.97	10464.28	10365.89	10335.94	1.000
3-Cluster	-3641.34	7540.55	7407.25	7366.67	1.000
4-Cluster	-3317.20	6959.81	6791.60	6740.39	0.999

Note: LL, Log Likelihood; BIC, Bayesian Information Criterion; aBIC, Sample-Size Adjusted BIC; AIC, Akaike Information Criterion.

**Table 2:** Comparison of the percentage of covariates between clusters (n, %).

Covariates	Lower residual (n=290)	Higher residual (n=174)	*P*
*Age(mean±sd)*	50.57±11.10	47.24±9.62	.001
*Gender *(n, %)			.755
Male	144(49.66)	89(51.15)	
Female	146(50.34)	85(48.85)	
*Number of family members *(n, %)			.627
≤2	34(11.72)	18(10.34)	
3	46(15.86)	23(13.22)	
≥4	210(72.41)	133(76.44)	
*Ethnicity *(n, %)			<0.001
Han	238(82.07)	164(94.25)	
Hui	52(17.93)	10(5.75)	
*Education level *(n, %)			.056
No formal school education	92(31.72)	38(21.84)	
Primary school	92(31.72)	52(29.89)	
Junior high school	84(28.97)	67(38.51)	
High school and above	22(7.59)	17(9.77)	
*Marital status *(n, %)			<0.001
Married	283(97.59)	153(87.93)	
Unmarried/Others	7(2.41)	21(12.07)	
*Income group *(n, %)			.001
≤3000 CNY	91(31.38)	26(14.94)	
3001-10000 CNY	73(25.17)	48(27.59)	
10001-20000 CNY	65(22.41)	54(31.03)	
≥20001 CNY	61(21.03)	46(26.44)	
*Disease *(n, %)			<0.001
No	208(71.72)	155(89.08)	
Yes	82(28.28)	19(10.92)	
*Smoking status *(n, %)			.032
Every day	79(27.24)	65(37.36)	
Smoking, but not every day	11(3.79)	1(0.57)	
Former smoker, now quit	19(6.55)	9(5.17)	
Never	181(62.41)	99(56.90)	
*Drinking status *(n, %)^a^			.037
Seldom	45(15.52)	43 (24.71)	
Often	69(23.79)	32(18.39)	
Never	176(60.69)	99(56.90)	
*Physical excise habit *(n, %)			<0.001
No	223(76.90)	164(94.25)	
Yes	67(23.10)	10(5.75)	
*Breakfast frequency *(n, %)			.149
Almost everyday	161(55.52)	115(66.09)	
Occasionally	58(20.00)	27(15.52)	
Few	27(9.31)	14(8.05)	
Never	44(15.17)	18(10.34)	
*Salt *(n, %)			.035
Lighter	123(42.41)	53(30.46)	
Moderate	99(34.14)	74(42.53)	
Heavier	68(23.45)	47(27.01)	
*Village *(n, %)			<0.001
Maosheng	84(28.97)	65(37.36)	
Yinhe	95(32.76)	25(14.37)	
Heshun	75(25.86)	58(33.33)	
Wudu	36(12.41)	26(14.94)	

Note: a: often represents having drinking behaviour within the last 30 days; seldom means having drinking behaviour over 30 days; never depicts participants who refused drinking anytime.

**Table 3:** Comparison of the transfer score of SRHMS dimension between clusters (mean±sd).

Dimension	Lower residual (n=290)	Higher residual (n=174)	*P*
B1	67.29±14.96	68.58±13.19	0.350
B2	93.79±16.52	92.33±16.39	0.345
B3	87.79±20.33	90.71±15.67	0.106
BZT	81.11±13.21	82.08±11.56	0.435
M1	68.96±23.03	60.32±22.54	<0.001
M2	80.52±16.67	83.83±12.38	0.025
M3	65.76±22.73	71.51±16.12	0.004
MZT	72.17±15.92	70.39±13.18	0.215
S1	81.96±14.45	79.66±12.42	0.081
S2	69.44±20.88	74.28±12.14	0.006
S3	69.62±20.62	75.65±15.82	0.001
SZT	73.64±15.34	76.41±9.30	0.031
SCZT	76.04±11.36	76.55±7.58	0.597

Note: B1, B2, and B3 were used to describe the physical symptoms and organic functions, daily physical activities, and physical mobility, respectively; M1, M2, and M3 were used to represent psychosocial symptoms and negative emotions, positive emotions, and cognitive functions, respectively; S1, S2, and S3 were used to depict role activity and social adaptability, social resources and social contacts, and social support, respectively.

**Table 4:** Associations between vegetable pesticide residual latent clusters and SRH and each dimension (*β*, 95% CI).

Model	B1	B2	B3	BZT	M1	M2	M3	MZT	S1	S2	S3	SZT	SCZT
Model0	1.54	-0.61	3.37	1.50	-7.56	4.62	6.43	-0.55	-1.08	5.73	6.03	3.71	1.47
	(-1.15,4.25)	(-3.59,2.37)	(-0.14,6.88)	(-0.82,3.82)	(-11.87, -3.26)	(1.80,7.44)	(2.56,10.30)	(-3.33,2.22)	(-3.63,1.46)	(2.33,9.14)	(2.47,9.59)	(1.19,6.22)	(-0.38,3.32)
Model1	0.84	-0.77	2.13	0.78	-7.89	3.82	4.44	-1.37	-2.16	4.46	6.36	2.81	0.65
	(-1.94,3.62)	(-3.84,2.30)	(-1.40,5.65)	(-1.58,3.15)	(-12.32, -3.47)	(0.94,6.70)	(0.56,8.31)	(-1.20, 1.46)	(-4.71,0.38)	(1.00,7.92)	(2.66,10.07)	(0.25,5.36)	(-1.21,2.52)
Model2	1.91	-1.25	2.53	1.23	-8.86	3.73	5.40	-1.54	-1.75	5.28	5.30	3.01	0.87
	(-0.85,4.67)	(-4.29,1.80)	(-1.05,6.11)	(-1.14,3.61)	(-13.22, -4.50)	(0.86,6.60)	(1.49,9.32)	(-4.36,1.28)	(-4.35,0.85)	(1.80,8.76)	(1.72,8.88)	(0.45,5.57)	(-1.01,2.76)
Model3	1.71	-1.17	3.12	1.34	-9.38	4.30	6.19	-1.56	-1.87	7.23	6.85	4.29	1.20
	(-1.08,4.50)	(-4.22,1.88)	(-0.49,6.73)	(-1.05,3.73)	(-13.74, -5.03)	(1.40, 7.21)	(2.24,10.15)	(-4.37,1.24)	(-4.48,0.75)	(3.76,10.71)	(3.19,10.52)	(1.71, 6.87)	(-0.70, 3.10)
Model4	1.15	-0.94	3.17	1.16	-8.66	4.19	5.74	-1.37	-1.68	5.30	5.94	3.31	0.94
	(-1.60,3.90)	(-3.98,2.09)	(-0.41,6.75)	(-1.20,3.52)	(-13.03, -4.30)	(1.32, 7.06)	(1.80, 9.68)	(-4.17,1.44)	(-4.27,0.90)	(1.83, 8.77)	(2.31,9.57)	(0.74,5.87)	(-0.93,2.82)
Model5	0.45	-1.16	2.42	0.43	-8.82	4.20	4.94	-1.34	-1.31	6.23	6.06	3.92	0.88
	(-2.15,3.05)	(-4.20,1.87)	(-1.13,6.00)	(-1.90,2.75)	(-13.13, -4.50)	(1.33,7.07)	(1.13,8.74)	(-4.15,1.47)	(-3.91,1.28)	(2.77,9.70)	(2.49,9.64)	(1.36,6.48)	(-1.00,2.75)
Model6	-0.01	-2.42	0.57	-0.57	-12.04	2.21	2.62	-4.37	-3.69	5.77	6.30	2.84	-0.81
	(-2.84,2.82)	(-5.67,0.84)	(-3.15,4.30)	(-3.04,1.91)	(-16.63, -7.46)	(-0.85,5.27)	(-1.41,6.64)	(-7.30, -1.44)	(-6.41, -0.97)	(2.11,9.42)	(2.40,10.19)	(0.12,5.56)	(-2.77,1.15)

Model0, empty model, latent cluster set as independent variable; Model1, add gender, ethnicity, age, education, marital status, and number of family members in model0; Model2, add income group based on Model0; Model3, smoking status, drinking habit, frequent physical exercise, frequent breakfast, and salt were added in Model0; Model4, disease status was added in Model0; Model5, adjusted survey year in the model0; Model6, adjusted all of covariates in the model.

B1: physical symptom and organic function; B2: daily physical activities; B3: physical mobility; M1: psychosocial symptom and negative emotion; M2: positive emotion; M3: cognitive function; S1: role activity and social adaptability; S2: social resource and social contact; S3: social support; BZT: physical health; MZT: mental health; SZT: social health; SCZT: total score of SRHMS.

## Data Availability

All relevant data are contained within the paper. Additional information can be obtained by contacting Dr. Jiangping Li (jpingl1273@163.com).

## Supplementary Materials

Supplementary Materials Table S1: instrument list was used in experimental in this study. Table S2: reagent list was used in experimental in this study. Table S3: questionnaire of SRHMS score assignment and dimensions, subscale score calculated. Table S4: the number and percentage of cases in different cluster for vegetable pesticide. Table S5: SRMHS different dimension score in raw and transfer status among greenhouse farmers from Yinchuan, China. Table S6: sensitivity analysis results. Figure S1: cluster-specific probabilities of vegetable pesticide residual for the three-cluster model (n=464, farmers from vegetable greenhouse in Yinchuan, China).
