# Supplementary material for: Comparison of Self-Rated Health among Characteristic Groups of Vegetable Greenhouse Farmers Based on Exposure to Pesticide Residuals: A Latent Profile Analysis
Source: Biomed Res Int. 2019 Apr 4;2019:2518763. doi: 10.1155/2019/2518763 (PMC6475569; doi:10.1155/2019/2518763)
Supplement: Supplementary Materials — Table S1: instrument list was used in experimental in this study. Table S2: reagent list was used in experimental in this study. Table S3: questionnaire of SRHMS score assignment and dimensions, subscale score calculated. Table S4: the number and percentage of cases in different cluster for vegetable pesticide. Table S5: SRMHS different dimension score in raw and transfer status among greenhouse farmers from Yinchuan, China. Table S6: sensitivity analysis results. Figure S1: cluster-specific probabilities of vegetable pesticide residual for the three-cluster model (n=464, farmers from vegetable greenhouse in Yinchuan, China). [file 2518763.f1.doc]

Supplementary File

Table S1. List of instruments used in this study

| Instrument | Model | Manufacturer |
| --- | --- | --- |
| GC-MSGC-MS | QP2010 | Shimadzu corporation of Japan |
| Autosampler | 7683series | Aglient inc, America |
| Chromatographic column | DB-1701 | Aglient inc, America |
| High purity helium | ≥99.999 | Jining Xieli special co. LTD, China |
| High purity nitrogen | ≥99.999 | Jining Xieli special co. LTD, China |
| Rotary evaporator | RE-52AA | Shanghai yirong biochemical instrument factory, China |
| Juicer | XB-9146F | Guangdong Xinbao Electrical Appliances Holdings Co.,Ltd, China |
| High speed homogenizer | PRO250 | PRO Scientific inc. America |

Table S2. List of reagents were used in this study

| Reagent | Specification | Manufacturer |
| --- | --- | --- |
| Sodium chloride | 140℃ bake 4h | Tianjin Damao chemical reagent plant, China |
| Sodium sulphate anhydrous | 650℃ bake 4h | Tianjin Damao chemical reagent plant, China |
| Normal hexane | Chromatographically pure | Fisher Scientific, America |
| Acetonitrile | Chromatographically pure | Fisher Scientific, America |
| Methyl alcohol | Chromatographically pure | Fisher Scientific, America |
| Organic microporous membrane | 0.45μl | Tianjin pilotage experimental equipment co. LTD, China |
| Pesticide standard solution | 100μl/ml | Shanghai pesticide research institute co. LTD, China |
| CARB/NH2 | 500mg/500mg/ml | Shanghai Hamu instrument technology co. LTD, China |

Table S3. Questionnaire for assignment and dimensions SRHMS scores and calculation of subscale scores

| Dimension | Number of items | Label | Re-score | Score of dimensions | Subscale score | Total of scale |
| --- | --- | --- | --- | --- | --- | --- |
| Physical symptom and organic function | 7 | B1 | Positive assignments items 1,2,3,6  Negative assignments items 4,5,7 | 1+2+3+4+5+6+7 | Physical health  1+2+3+4+5+6+7+  8+9+10+11+12+  13+14+15+16+17 | SRHMS  1+2+3+4+5+6+7+  8+9+10+11+12+  13+14+15+16+17+  19+20+21+22+23+  24+25+26+27+28+  29+30+31+32+33+  35+36+37+38+39+  40+41+42+43+44+  45+46 |
| Daily physical activities | 5 | B2 | Positive assignments items 8,9,10,11,12 | 8+9+10+11+12 |
| Physical mobility | 5 | B3 | Positive assignments items 13,14,15,16,17 | 13+14+15+16+17 |
| Positive emotion | 5 | M2 | Positive assignments items 19,20,21,22,23 | 19+20+21+22+23 | Mental health  19+20+21+22+23+  24+25+26+27+28+29  +30+31+32+33 |
| Psychosocial symptom and negative emotion | 7 | M1 | Negative assignments items 24,25,26,27,28,29,30 | 24+25+26+27+28+29+30 |
| Cognitive function | 3 | M3 | Positive assignments items 31, 32, 33 | 31+32+33 |
| Role activity and social adaptability | 4 | S1 | Positive assignments items 35, 36, 37 38 | 35+36+37+38 | Social health  35+36+37+38+  39+40+41+42+43+  44+45+46 |
| Social resource and social contact | 5 | S2 | Positive assignments items 39, 40, 41, 42, 43 | 39+40+41+42+43 |
| Social support | 3 | S3 | Positive assignments items 44, 45, 46 | 44+45+46 |

Table S4. Number and percentage of cases in different clusters for vegetable pesticide

| Model | Cluster1 | Cluster2 | Cluster3 | Cluster4 |
| --- | --- | --- | --- | --- |
| 1-Cluster | 464(100) |  |  |  |
| 2-Cluster | 290(62.50) | 174(37.50) |  |  |
| 3-Cluster | 285(61.42) | 5(1.08) | 174(37.50) |  |
| 4-Cluster | 5(1.08) | 100(21.53) | 285(61.42) | 74(15.97) |


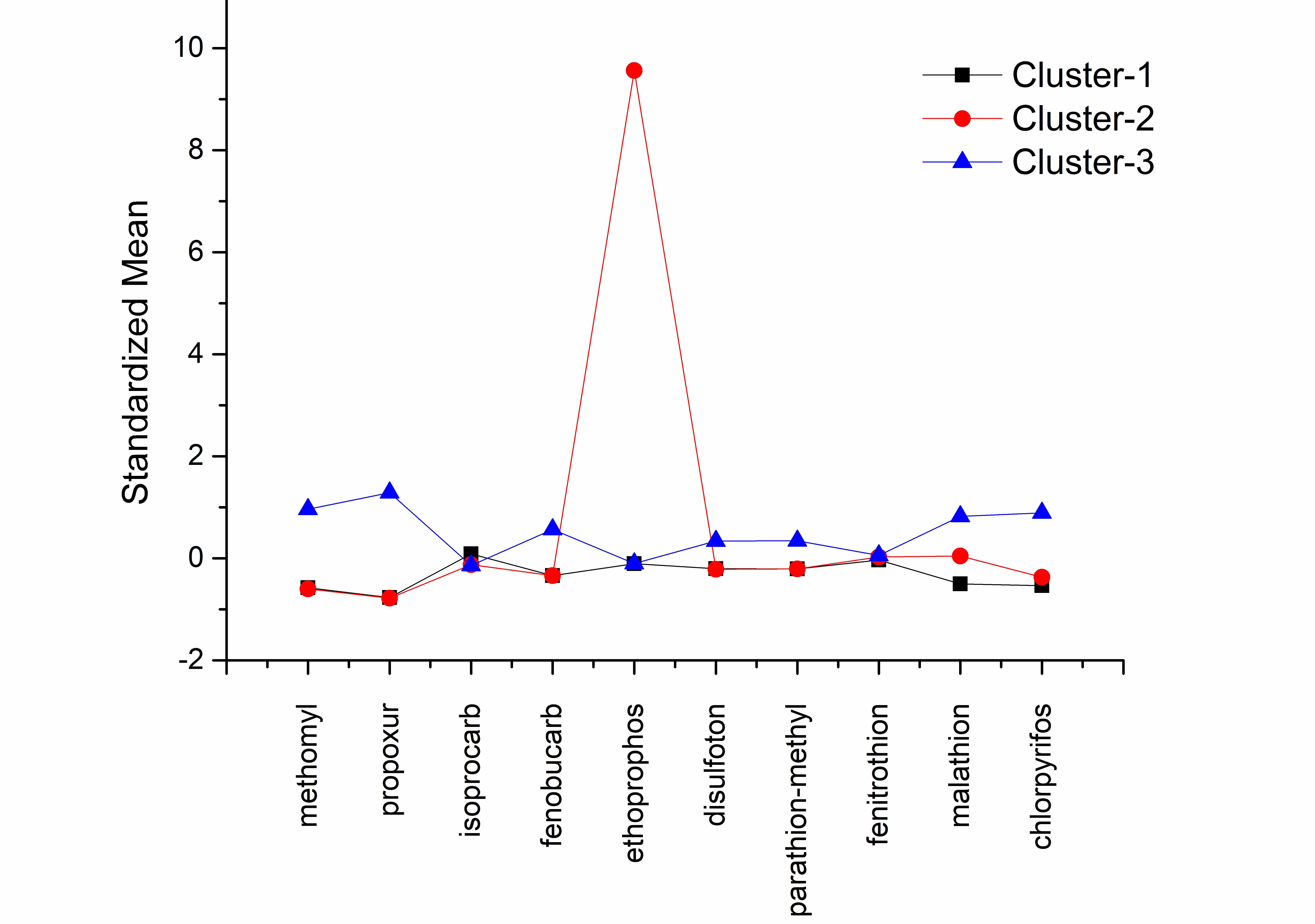


Fig S1. Cluster-specific probabilities of vegetable pesticide residual for the three-cluster model (n=464, farmers from vegetable greenhouse in Yinchuan, China)

Table S5. SRMHS score for different dimensions in raw and transfer status among greenhouse farmers from Yinchuan, China

| Dimension | RAW SCORE | | | |  | TRANSFERMER SCORE | | | |
| --- | --- | --- | --- | --- | --- | --- | --- | --- | --- |
|  | Min | Max | Mean | SD |  | Min | Max | Mean | SD |
| B1 | 15 | 69 | 47.44 | 10.02 |  | 21.43 | 98.57 | 67.77 | 14.32 |
| B2 | 0 | 50 | 46.62 | 8.23 |  | 0.00 | 100.00 | 93.25 | 16.47 |
| B3 | 0 | 50 | 44.44 | 9.38 |  | 0.00 | 100.00 | 88.89 | 18.75 |
| BZT | 37 | 169 | 138.51 | 21.45 |  | 21.76 | 99.41 | 81.47 | 12.61 |
| M1 | 2 | 70 | 46.00 | 16.24 |  | 2.86 | 100.00 | 65.72 | 23.20 |
| M2 | 5 | 50 | 40.88 | 7.64 |  | 10.00 | 100.00 | 81.76 | 15.28 |
| M3 | 0 | 30 | 20.38 | 6.20 |  | 0.00 | 100.00 | 67.92 | 20.67 |
| MZT | 18 | 149 | 107.26 | 22.44 |  | 12.00 | 99.33 | 71.50 | 14.96 |
| S1 | 4 | 40 | 32.44 | 5.50 |  | 10.00 | 100.00 | 81.09 | 13.75 |
| S2 | 0 | 50 | 35.63 | 9.12 |  | 0.00 | 100.00 | 71.25 | 18.24 |
| S3 | 2 | 30 | 21.56 | 5.75 |  | 6.67 | 100.00 | 71.88 | 19.17 |
| SZT | 12 | 120 | 89.62 | 16.12 |  | 10.00 | 100.00 | 74.69 | 13.44 |
| SCZT | 124 | 431 | 335.41 | 44.41 |  | 28.18 | 97.95 | 76.23 | 10.09 |

Table S6. Sensitivity analysis results

| Stratify | BZT | M1 | MZT | S1 | S2 | S3 | SZT | SCZT |
| --- | --- | --- | --- | --- | --- | --- | --- | --- |
| ***Exposure time <200 days*** | | | | | | | | |
| Model6 | -2.49  (-11.14,6.15) | -11.81  (-22.66, -0.96) | -5.34  (-12.80,2.11) | -11.63  (-19.20,-4.06) | -1.02  (-11.03, 9.00) | -7.73  (-19.52,4.05) | -5.83  (-13.64,1.98) | -4.33  (-9.66,0.99) |
| ***Exposure time 200-299 days*** | | | | | | | | |
| Model6 | -2.56  (-7.04,1.93) | -16.06  (-24.85, -7.28) | -7.18  (-13.00, -1.35) | -6.39  (-11.67,-1.11) | -0.73  (-8.42,6.96) | 0.62  (-5.75,7.00) | -2.26  (-7.48,2.96) | -4.04  (-7.84,-0.24) |
| ***Exposure time ≥300 days*** | | | | | | | | |
| Model6 | -2.44  (-0.80,5.67) | -9.60  (-15.75, -3.45) | -1.77  (-5.63, 2.08) | -0.90  (-4.36, 2.56) | 9.44  (4.50,14.39) | 9.94  (4.68,15.21) | 6.12  (2.46, 9.78) | 2.02  (-0.60,4.65) |
